# A novel approach to the Orienteering Problem based on the Harmony Search algorithm

**DOI:** 10.1371/journal.pone.0264584

**Published:** 2022-02-28

**Authors:** Krzysztof Szwarc, Urszula Boryczka

**Affiliations:** Institute of Computer Science, University of Silesia in Katowice, Sosnowiec, Poland; Torrens University Australia, AUSTRALIA

## Abstract

This article presents a new approach to designing a Harmony Search (HS) algorithm, adapted to solve Orienteering Problem (OP) instances. OP is a significant NP-hard problem that has considerable practical application, requiring the development of an effective method for determining its solutions. The proposed HS has demonstrated its effectiveness through determined optimum results for each task from the six most popular benchmarks; a marginal number approximated the best results, with the average error below 0.01%. The article details the application of this described algorithm, comparing its results with those of state-of-the-art methods, indicating the significant efficiency of the proposed approach.

## 1 Introduction

The Orienteering Problem (OP) is an NP-hard [1] optimization problem that assumes the need to maximising the number of points received due to visiting particular nodes within a pre-defined time limit. According to [2], this can be considered a combination of two issues that are significant from a utilitarian perspective: the Knapsack Problem and the Traveling Salesman Problem. Connecting features of these problems enables application of the OP in economic practice and optimizes many processes occurring in the logistics area of interest, such as planning a traveling salesman route, where time limits prevent the salesman from visiting all of the towns [3], solving tourist–route design problems [4], and analyzing other inventory or routing problems [1]. It should also be emphasized that one OP variant is distinct from the utilitarian persperctive beacuse it includes *M* of team members (i.e., the Team Orienteering Problem (TOP)). This is among the Vehicle Routing Problems with Profits (VRPP) variants [5] that are characterized by the need to select a subset of customers, describe them in terms of profit, and design routes according to which the sum of the profits gained is maximized under time (distance) constraints. Among these variants, we can also distinguish the Capacitated Profitable Tour Problem (assuming maximization of profits minus the travel costs under capacity restrictions) and a variant featuring a private fleet and common carrier, in which some customers may be delegated to another carrier subject to a cost [5].

**Fig 1:**
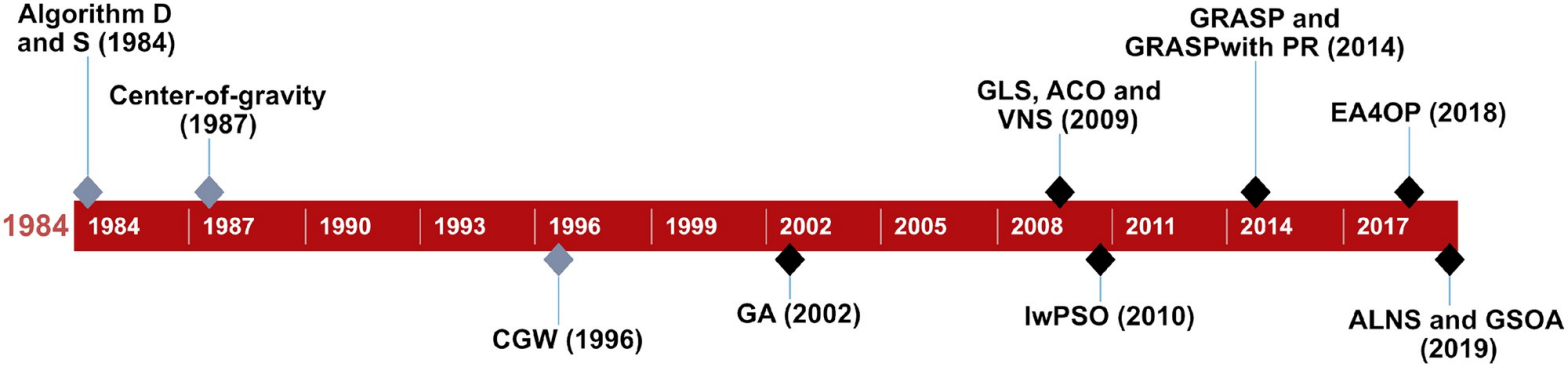
The chronology of selected publications referring to the adjustment of techniques to solve the OP.

Because this issue pertains to the class of NP-hard problems, approximate approaches are often applied to solve OP instances, precluding the design of optimal travel routes within an acceptable timeframe (according to [2], using exact algorithms is too time-consuming for the OP to be applied in practice). Among the existing heuristics used to address the analyzed problem, those worth emphasising are the deterministic D algorithm [3], stochastic S algorithm [3], center-of-gravity [1], and CGW algorithm proposed in [6]. More up-to-date approaches to the OP assume the use of metaheuristics, which draw inspiration from nature. Tasgetiren achieved good solutions using the Genetic Algorithm with an Adaptive Penalty Function (GA) [7]. Vansteenwegen et al. tested the Guided Local Search (GLS) [8], Schilde et al. adjusted the Pareto Ant Colony Optimization (ACO) and Pareto Variable Neighborhood Search (VNS) [4], Sevkli and Sevilgen used different variants of Particle Swarm Optimization (e.g., the Intensification Strengthened PSO (IS-PSO)) [9], Campos et al. checked the efficiency of the Greedy Randomized Adaptive Search Procedure (GRASP) and the version making use of Path Relinking (GRASP with PR) [10], Kobeaga et al. proposed EA4OP [11], Santini adjusted the Adaptive Large Neighborhood Search (ALNS) [12], and Faigl analyzed the Growing Self-Organizing Array (GSOA) [13]. Fig 1 presents the chronology of the selected publications, indicating the adjustments to techniques to solve the OP. Meanwhile, more recent approaches to solving selected OP variants include the adaptation of the VNS [14] and a Biased Random-Key Genetic Algorithm [15] for the Set OP (a generalization of the OP), the proposal to apply the GSOA to the Close-Enough Traveling Salesman Problem [16], adaptation of the Tabu Search to the Probabilistic Orienteering Problem [17], solving OP with hotel selection by the Ant Colony System [18], and the use of Max–Min Ant Colony Optimization to solve the Thief OP instances [19].

Given the multiplicity of NP-hard problems modeling various utilitarian issues, new metaheuristics have been created, enabling the discovery of suboptimal solutions within an acceptable timeframe. Among the relatively new methods characterized by significant efficiency is the Harmony Search (HS). This technique was proposed in [20] and successfully applied to a water pump switching problem [21], the Sudoku solving process [22], the design of steel frames [23], university course timetabling [24], route optimization for on-demand e-waste collection [25], nurse rostering problems [26], microstructural image classification [27], coordination of drug transportation [28], frequency stabilization in interconnected power system [29], and the Asymmetric Traveling Salesman Problem (ATSP) [30].

According to the no free lunch theorem [31], there is no single metaheuristic that can solve all existing optimization problems in a better way than other methods. For this reason, researchers in the field of computational intelligence are forced to empirically verify the effectiveness of a given method for a specific optimization problem in order to determine its usefulness.

This article aims to eliminate the research gap surrounding adjusting HS to successfully solve OP instances. We believe that the HS’s significant efficiency, which has been noted in the context of solving similar optimization problems, can enable the development of a method that can construct more favorable solutions than other metaheuristics commonly used to solve OP instances. In this way, we want to contribute by proposing a method that can be successfully used in business practice to solve large OP instances, the complexity of which makes it impossible to apply exact algorithms.

Among the research adapting HS to solve various OP variants, we can distinguish the paper [32] and [33], which propose variants of the algorithm designed to approximate the Generalized Orienteering Problem. Elsewhere, Tsakirakis et al. has described an interesting Similarity Hybrid HS algorithm for the TOP [34]. However, this paper’s approach is based on the idea proposed in [30], which achieved satisfactory results for a similar optimization problem (i.e., the ATSP).

This article comprises seven sections. After the introduction to the subject matter, the HS’s characteristic features are presented. Next, we describe the OP and present the HS structure as adjusted to solve the OP. After explaining the research methodology in more detail, we discuss the results obtained. The paper the details the study’s conclusions and plans for further research.

The results presented in the article have been published in the doctoral dissertation [35].

## 2 Description of the classical Harmony Search

The HS is a modern metaheuristic proposed in [20] and based on the similarity of musical improvisation to the search for the optimum in optimization processes. The algorithm’s author transcribed real-world observations onto the HS diagram by implementing the following parameters and structures:

- Pitch: an element that corresponds to the decision variable of the given optimization problem.
- Harmony: a structure comprising *n* pitches (where *n* represents the number of decision variables describing the given problem) and which is a complete solution to the problem.
- *HM* (Harmony Memory): a structure storing harmonies sorted according to their objective function value (the worst element is positioned last).
- *HMS* (Harmony Memory Size): the size of the *HM* (i.e., the number of harmonies stored in the *HM*).
- *HMCR* (Harmony Memory Consideration Rate): the probability of playing the pitch by heart. With a probability equal to the *HMCR*, the choice of pitch value takes place in position *i* in the created solution, based on the value of pitches in position *i*, describing harmonies stored in *HM*. With a probability of 1 − *HMCR* the random value of pitch *i* is selected from the acceptable range.
- *PAR* (Pitch Adjustment Rate): the probability of modification of the pitch value in the *i* position on the basis of the value of pitches in position *i* in *HM*.
- *bw* (Bandwidth): the range of pitch value changes relative to the parameter *PAR*, with a value corresponding to the maximum change value of the chosen pitch in position *i*.
- *IT*: it defines the condition in which the algorithm stops (interpreted as the number of harmonies created in the main method loop).

At the beginning of an HS operation, *HMS* harmonies are created and, included in the *HM*. They are subsequently sorted based on objective function value, such that the worst harmony is found in the last position.

According to [36], improvisation in music assumes the aim of better harmonization involves trying various pitch combinations according to the following possibilities:

1. Playing the pitch by heart.
2. Modifying the pitch by heart.
3. Playing a random pitch from within the acceptable range.

Based on music’s improvisation process, Geem assumed that the algorithm must iteratively create subsequent harmonies, choosing the value of the next pitch *i* according to the following rules:

1. The choice of pitch *i*, based on the value in position *i* in *HM* (probability equals *HMCR* ⋅ (1 − *PAR*)).
2. The choice of modified pitch *i*, based on the value in position *i* in *HM* (probability equals *HMCR* ⋅ *PAR*).
3. The choice of pseudorandom value from the available range (probability equals 1 − *HMCR*).

**Fig 2:**
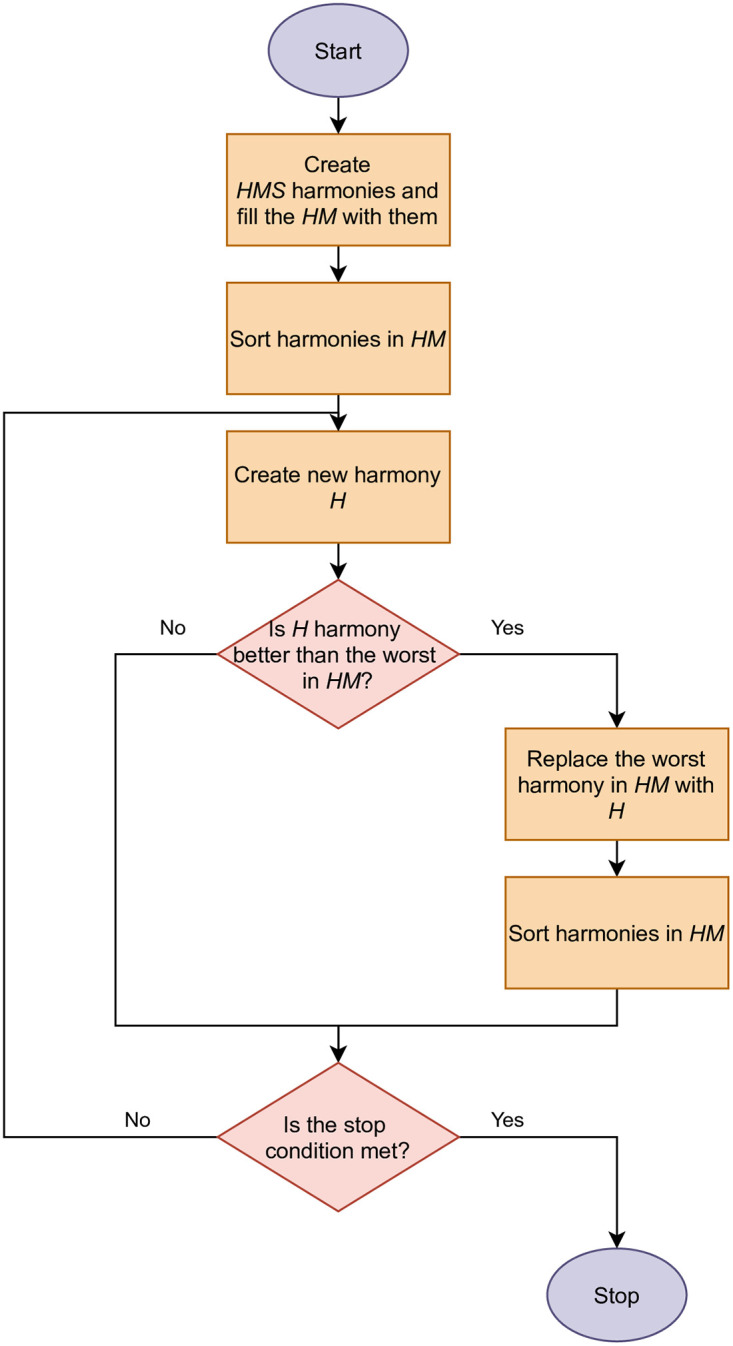
Flowchart of the HS.

A new harmony’s objective function value is compared with the applicable value describing the worst element located in the *HM*. If the new solution is more beneficial, it replaces the worst harmony stored in the *HM*, after which the elements of the *HM* are sorted again. The procedure is repeated for *IT* iterations. Fig 2 presents a flowchart of the HS.

However, the HS has caused considerable controversy within the research community. For example, according to Weyland, the algorithm is simply represents a special case of the Evolution Strategies (ES) and does not offer any novelty [37]. The author conducted a detailed analysis to demonstrate the imperfections in the results presented by Geem in [22]. However, in [38] Kim responded Weyland’s allegations by demonstrating the differences between the HS and ES.

## 3 Formulation of the Orienteering Problem

This article is based on the formulation of the OP presented by Vansteenwegen et al. [2] and assumes the occurrence of a set of *N* nodes and assigns a number of points *S*_*i*_ to each *i* node. After stablishing the beginning node (marked 1), ending node (marked *N*), travel time between nodes *i* and *j* (for all nodes), *t*_*ij*_, and the time limit *T*_*max*_ are set, the decision variables are introduced: *x*_*ij*_ (which assumes a value of 1 if node *j* is visited after node *i* and 0 otherwise) and *u*_*i*_ (which indicates the position of node *i* in the path).

The objective function of the problem, assuming the maximization of collected points, is determined according to the following:

$$\begin{array}{c} max\sum_{i=2}^{N-1}\sum_{j=2}^{N}S_{i}x_{ij}. \end{array}$$ (1)

The limiting condition, ensuring that the path starts with Node 1 and ends at node *N*, is as follows:

$$\begin{array}{c} \sum_{j=2}^{N}x_{1j}=\sum_{i=1}^{N-1}x_{iN}=1. \end{array}$$ (2)

The condition enforcing the creation of connections in the path and guaranteeing that each node is not visited more than once is determined according to the following:

$$\begin{array}{c} \sum_{i=1}^{N-1}x_{ik}=\sum_{j=2}^{N}x_{kj}\leq 1;\;\forall k=2,\ldots ,N-1. \end{array}$$ (3)

The need to comply with the time limit *T*_*max*_ is determined as follows:

$$\begin{array}{c} \sum_{i=1}^{N-1}\sum_{j=2}^{N}t_{ij}x_{ij}\leq T_{max}. \end{array}$$ (4)

To eliminate subtours, the following is introduced:

$$\begin{array}{c} 2\leq u_{i}\leq N;\;\forall i=2,\ldots ,N, \end{array}$$ (5)

$$\begin{array}{c} u_{i}-u_{j}+1\leq \left(N-1\right)\left(1-x_{ij}\right);\;\forall i,j=2,\ldots ,N. \end{array}$$ (6)

Additionally, the following is assumed:

$$\begin{array}{c} x_{ij}\in \left\{0,1\right\};\;\forall i,j=1,\ldots ,N. \end{array}$$ (7)

## 4 Harmony Search adjusted to solve the Orienteering Problem

**Fig 3:**
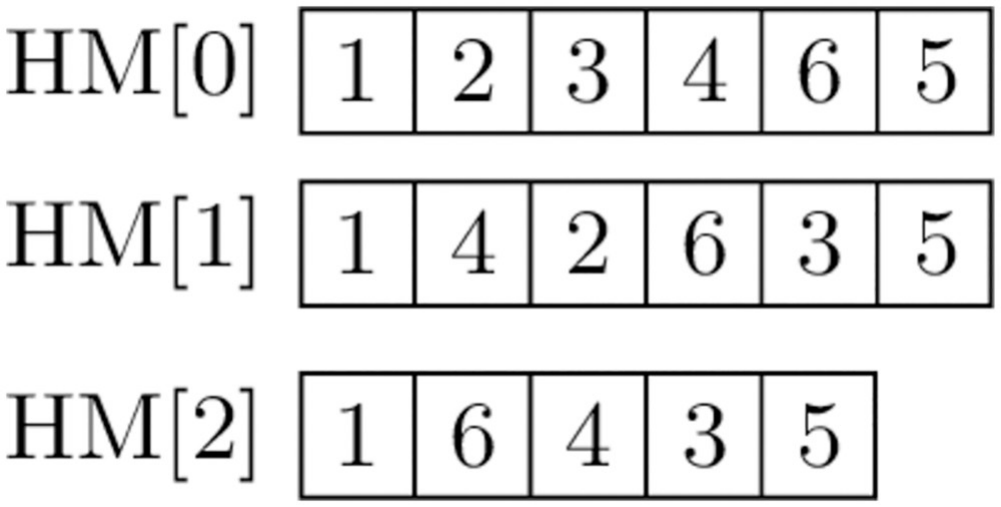
Content of *HM*.

The OP solution comprises a sequence of points that must be visited in a given order. Given the similarity between the OP and ATSP solutions, we decided that the natural representation of the *HM* would be the path to be visited, in the order in which individual pitches appear. Fig 3 presents example content of the *HM* for *HMS* = 3, starting with Node 1 and ending with Node 5. Given the time limitations resulting from defining the problem, the particular harmonies may comprise various numbers of pitches.

**Fig 4:**
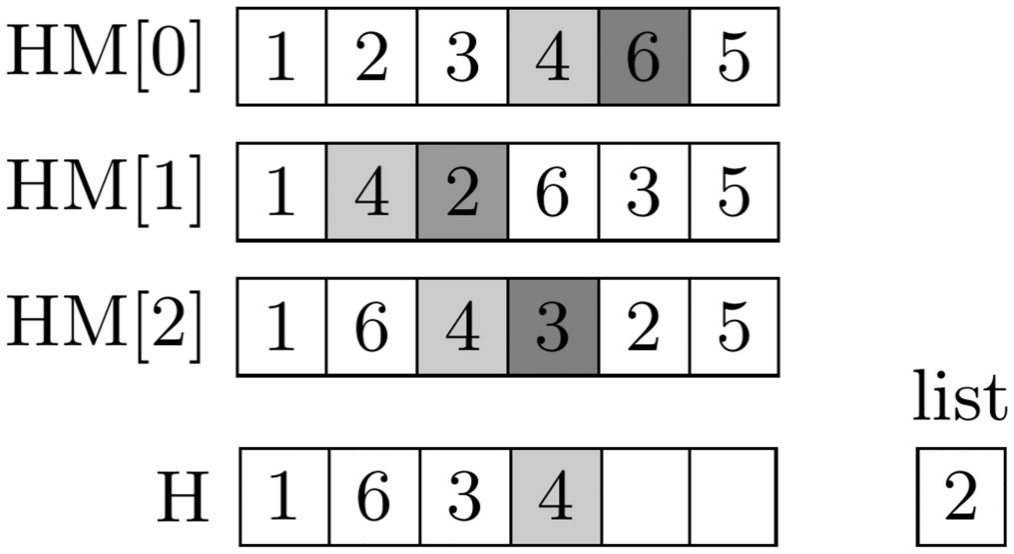
An example of generating the list of analysed nodes.

The process of choosing the value of the next pitch assumes the following:

1. Choosing pitch values is based on the elements of the *HM* (performed with the probability *HMCR* ⋅ (1 − *PAR*)), indicating that preparing the list of nodes, which occur in harmonies located in the *HM* directly after the last node added to a new harmony *H* (for the OP, as in the case of the ATSP, we assumed that it was more important to analyze the successors and predecessors of the node in the path than the absolute order in which the node was visited). In the example presented in Fig 4, the nodes occurring directly after Node 4 are analyzed. Although Node 6 and 3 appear in HM[0] and HM[2], they cannot be added to the new solution due to occuring in *H*. Only Node 2, which exists in HM[1], can potentially be added to the list created. However, before adding it to the list, it is necessary to check the possibility of managing it and, subsequently, point *N* (in example Node 5) without infringing on the limitation *T*_*max*_.
  Next, following the approach described by Komaki et al. [39], a node belonging to the list is chosen based on the objective function value of the harmony it derives from, using the roulette wheel selection method (thus increasing the method’s exploitation ability). In cases in which the list of nodes is empty, it is completed with the available nodes—i.e., the nodes not yet visited; after visiting, it will be possible to collect points in node *N*, considering the time limit *T*_*max*_—and by determining the quotient *g* for the number of points achieved (for each) by visiting the node compared to the distance (according to travel time) between the last visited node and the current one. The *HMS* nodes from the list, with the greatest *g* values subsequently, participate in the roulette wheel method selection (if the list contains fewer elements than *HMS*, all nodes are considered).
2. Modifying pitch value (executed with a probability equal to *HCMR* ⋅ *PAR*) is based on the modified heuristics proposed by Golden et al. [40] in an article including an analysis of the effect of individual component weights on heuristics efficiency that demonstrates how knowledge about the problem is used to increase the entire technique’s efficiency. In the HS discussed the designation for each available node *i* assumes the value:
  $$\begin{array}{c} W_{i}=0.7\cdot Rs_{i}+0.2\cdot Rc_{i}+0.1\cdot Rp_{i}, \end{array}$$ (8)
  where:
  - *Rs*_*i*_ ranks the number of points obtained at node *i* among the points describing available nodes.
  - *Rc*_*i*_ ranks the distance of node *i* (with coordinates (*x*_*i*_,*y*_*i*_)) from the center of gravity of available nodes (creating the set *A*), the coordinates of which derive from the formula:
    $$\begin{array}{c} \begin{array}{cc} x= & \frac{\sum_{i\in A}x_{i}\cdot S_{i}}{\sum_{i\in A}S_{i}}\\ y= & \frac{\sum_{i\in A}y_{i}\cdot S_{i}}{\sum_{i\in A}S_{i}} \end{array}. \end{array}$$ (9)
  - *Rp*_*i*_ ranks the distance of node *i* from the last visited node.
  Ranks are employed to limit the impact of various ranges of values of particular features describing nodes upon the value *W*_*i*_. It was assumed that ranks are given based on sorted values in the ascending order for *Rc*_*i*_ as well as *Rp*_*i*_—meaning the nearest nodes receive the rank 1, the next nearest receive the rank 2, and so on, and if two nodes are described by the same value, they receive the same rank, and the next node in line receives a rank that is increased by one—and in the descending order for *Rs*_*i*_, meaning rank 1 is assigned to the node(s) with the greatest number of points. Later ranks are normalized to one range for *Rc*_*i*_, *Rp*_*i*_, and *Rs*_*i*_.
  After receiving the values of *W*_*i*_ for each available node, the required pitch value is chosen by employing the roulette wheel method (promoting lower *W*_*i*_ values) for the maximum number of *HMS* nodes with the lowest value of *W*_*i*_.
3. Choosing (with a probability equal to 1-*HMCR*) an available pseudorandom node from the set of such nodes infers that it remains possible to visit the node *N* after visiting one of them without infringing on the time limit *T*_*max*_.

**Fig 5:**
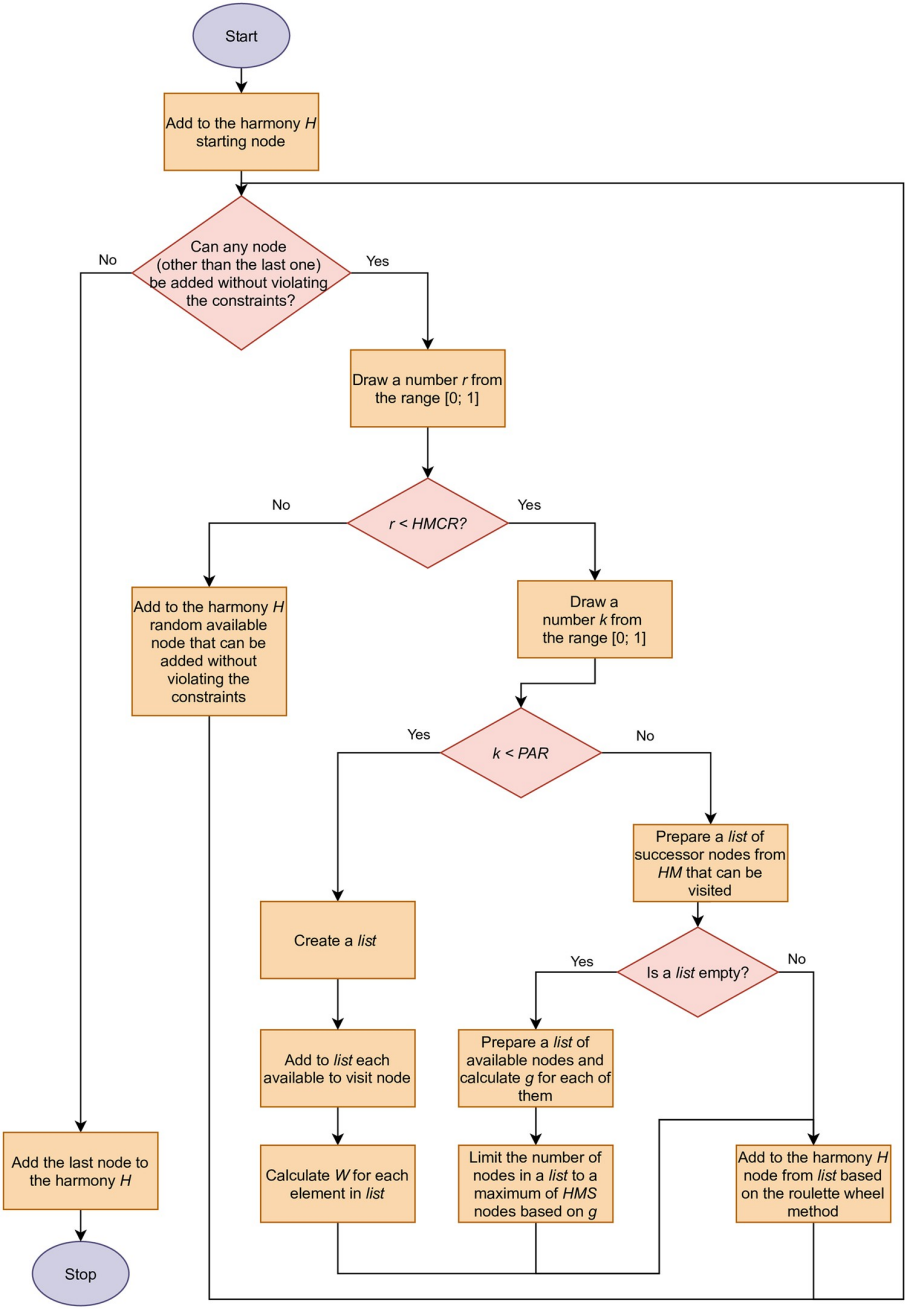
Flowchart of the process of selecting the value of the next pitch in the proposed HS.

Fig 5 presents a flowchart of the process of selecting the value of the next pitch in the propoesed HS.

After creating a new solution *H*, it is necessary to determine whether it is characterized by a more beneficial objective function value than the worst harmony stored in *HM*. In such cases, the procedure (based on the heuristics proposed by Golden et al. [1] and Chao et al. [6]) for improving it begins—building on the previous demonstration of the significant efficiency of this process at this location within the HS structure [41]—following four steps:

1. Optimizing a new route by means of the method 2-opt and using it to replace the worst solution located in *HM*.
2. Removing from the route node *i* (which cannot be the starting and ending node), which features the lowest value of the quotient *m*:
  $$\begin{array}{c} m=\frac{S_{i}}{matrix[p][i]+matrix[i][f]-matrix[p][f]}, \end{array}$$ (10)
  where:
  - *matrix*[*p*][*i*] represents the distance (travel time) between node *p* and *i*,
  - *p* is a node preceding node *i* in the analyzed route plan, and
  - *f* is a node that follows the node *i* in the analyzed route plan.
3. It is necessary to add as many available points as possible (for the best possible positions) to the route created in Step 2, beginning with those, whose appearance in the route enables the achievement of the largest possible value of quotient *m* (it is necessary to check value *m* for each point in each possible route position).
4. If the route was improved in Step 3 relative to the base solution, *H*, the plan needs to be optimized again using the 2-opt method, with this optimization replacing the solution added to *HM* in Step 1.

After executing *R* iterations from the last replacement of the worst solution, there is another draw of *HM* content (excluding the best solution) to avoid premature convergence. The efficiency of the mechanism for ATSP has previously been demonstrated in [42], indicating that by choosing the appropriate *R* value, we can preserve the diversity of *HM* content, maintaining an appropriate level of solution space exploartion.

**Fig 6:**
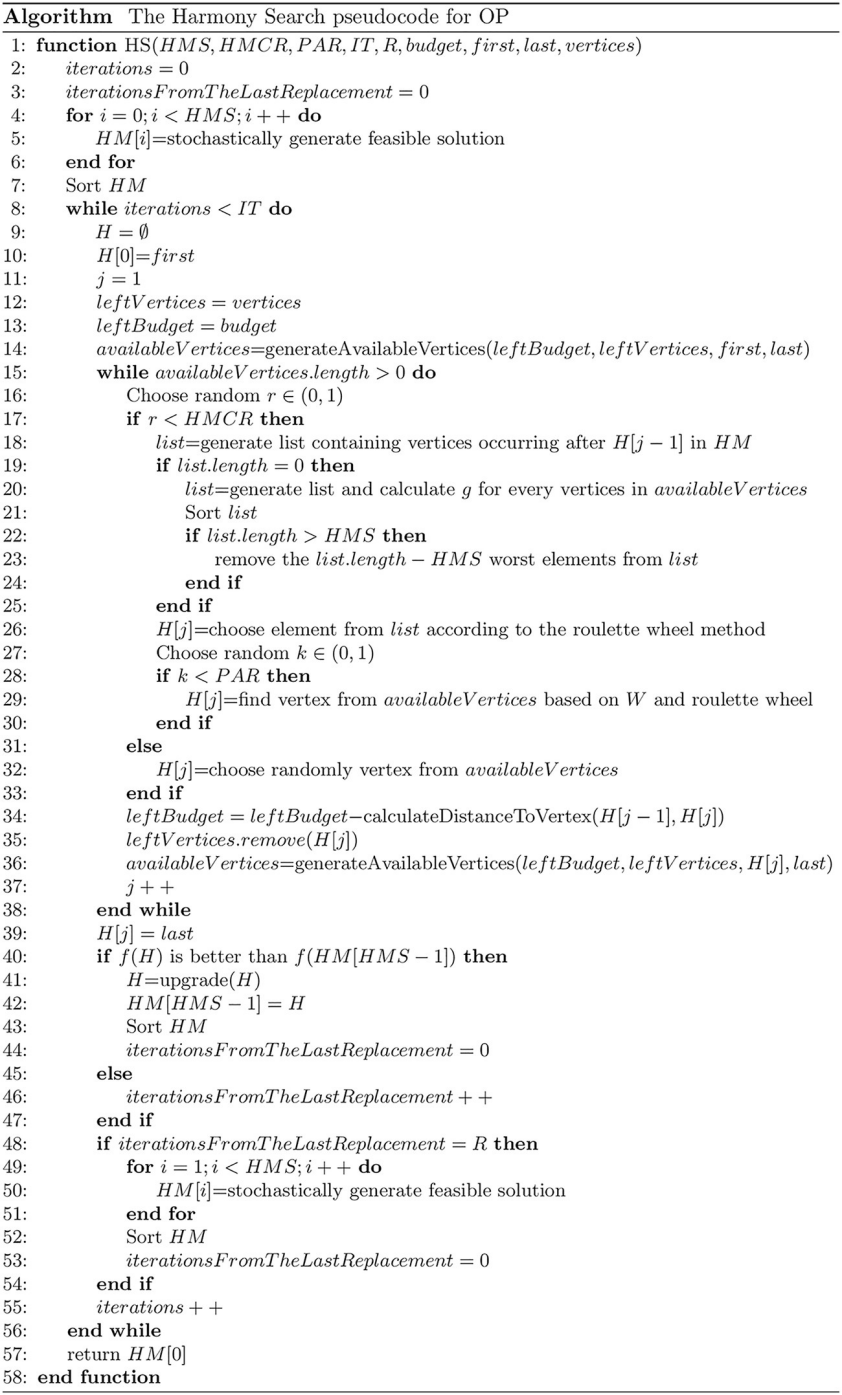
The pseudocode of HS adjusted to solve OP instances.

Fig 6 presents the HS pseudocode adjusted to solve OP instances.

## 5 Research methodology

The decision was made to use a ‘test bed’ in the form of four generally accessible sets of Tsiligirides tasks (where Set 4 is Set 1 with a correction based on [6]; the coordinates of one point (4.90, 18.90) were replaced with (4.90, 14.90)), Chao sets 64 and 66, and selected 45 tasks from Generation 1 of OPLib (analysed by [11]). Each task was solved 30 times using a different seed. Sets are available at [43] and [44].

Based on papers [30, 42, 45] and the performed empirical tests, the following values of HS parameters were determined: *HMCR* = 0.98, *PAR* = 0.1, *HMS* = 5, *IT* = 1000000 and *R* = 500.

The quality of solutions was assessed by measuring the number of iterations required to achieve convergence *it* (the average value was designated $\bar{it}$, and sample standard deviation as *σ*_*it*_), the time required to achieve convergence *t* [s] (the average value was designated as $\bar{t}$, the sample standard deviation as *σ*_*t*_), and the average error value as $\bar{e}$ (formula (11)), the best result’s error value *r* (formula (12)), and the worst (*w*) and the best (*b*) determined objective function values on the basis of 30 repetitions of the algorithm.

$$\begin{array}{c} \bar{e}=\frac{\left(optimum-average\right)}{optimum}\cdot 100\%. \end{array}$$ (11)

$$\begin{array}{c} r=\frac{\left(optimum-best\right)}{optimum}\cdot 100\%. \end{array}$$ (12)

The algorithm was implemented in *C*#, and the tests were performed using the laptop Lenovo Legion Y520, with the following components: Windows 10 Home 64-bit, 32 GB RAM (SO-DIMM DDR4, 2400MHz) and Intel Core i7-7700HQ (4 cores, 2.8 GHz to 3.8 GHz, 6 MB cache).

In order to verify the effectiveness of the hybridization of the HS algorithm with 2-opt, the tasks from the Tsiligirides and Chao sets were solved by means of a variant without the solution improvement step using 2-opt. The obtained mean value of the objective function for the variant without 2-opt was denoted as $\bar{e_{l}}$. Additionally, the obtained objective function values were subjected to the Wilcoxon Signed-Rank Test, using *R*. Both variants (noted as *V*1 and *V*2) were subjected to the process, whereas the value of 0.05 was assumed as the level of test significance (the obtained p-values described with the lower result indicate to accept the alternative hypothesis according to which *V*1 obtained greater results than *V*2).

The objective function values achieved by the HS were compared with the results obtained by the following algorithms according to the respective bibliographical sources:

- D algorithm [3],
- S algorithm [3],
- center-of-gravity [1],
- CGW [6],
- GA [7],
- GLS [8],
- ACO [4],
- VNS [4],
- IS-PSO [9],
- GRASP [10],
- GRASP with PR [10],
- GSOA [13],
- 2-Parameter IA [11],
- EA4OP [11].

## 6 Results

The results achieved by the HS are presented in Tables 1–7 for Tsiligirides sets 1, 2, 3 and 4 and Chao sets 64 and 66, and tasks from OPLib respectively. The attribute Best represents the best objective function value obtained in the papers reviewed (see, in particular [7, 8, 10, 11, 13]) and was considered to calculate the values *r* and *e*. These results support the significant efficiency of the proposed HS showing that it was able to determine the best routes for all 107 instances of the problem from Tsiligirides and Chao sets (*r* is equal to 0%).

**Table 1:** HS results for Tsiligirides 1.

*T*_*max*_	*r*	$\bar{e}$	$\bar{e_{l}}$	Best	*b*	*w*	$\bar{t}$	*σ*_*t*_	$\bar{it}$	*σ*_*it*_
5	0	0	0	10	10	10	<1	<1	1.00	0.00
10	0	0	0	15	15	15	<1	<1	1.20	0.76
15	0	0	0	45	45	45	<1	<1	130.60	275.57
20	0	0	0	65	65	65	<1	<1	3.93	4.44
25	0	0	0	90	90	90	<1	<1	1527.97	1658.44
30	0	0	0	110	110	110	<1	<1	30.97	96.15
35	0	0	0	135	135	135	<1	<1	392.13	669.92
40	0	0	0	155	155	155	<1	<1	4071.00	4601.66
46	0	0	0	175	175	175	<1	<1	232.23	313.19
50	0	0	0	190	190	190	<1	<1	175.53	389.27
55	0	0	0	205	205	205	4.30	4.38	21778.70	21893.81
60	0	0	0	225	225	225	2.60	3.30	12239.80	15478.65
65	0	0	0	240	240	240	<1	<1	938.77	1208.97
70	0	0	0	260	260	260	<1	<1	2162.10	3481.99
73	0	0	0	265	265	265	<1	<1	434.03	538.07
75	0	0	0	270	270	270	<1	<1	333.30	584.13
80	0	0	0	280	280	280	<1	<1	87.10	226.90
85	0	0	0	285	285	285	<1	<1	131.37	225.13
Average	0	0	0	167.78	167.78	167.78	0.49	0.57	2481.76	2869.28

**Table 2:** HS results for Tsiligirides 2.

*T*_*max*_	*r*	$\bar{e}$	$\bar{e_{l}}$	Best	*b*	*w*	$\bar{t}$	*σ*_*t*_	$\bar{it}$	*σ*_*it*_
15	0	0	0	120	120	120	<1	<1	30.60	66.34
20	0	0	0	200	200	200	<1	<1	7.73	10.50
23	0	0	0	210	210	210	<1	<1	965.60	710.26
25	0	0	0	230	230	230	<1	<1	2.17	1.56
27	0	0	0	230	230	230	<1	<1	1.57	0.90
30	0	0	0	265	265	265	<1	<1	5186.97	5303.63
32	0	0	0	300	300	300	<1	<1	405.17	517.76
35	0	0	0	320	320	320	<1	<1	2758.07	2961.46
38	0	0	0	360	360	360	<1	<1	772.07	1220.61
40	0	0	0	395	395	395	<1	<1	1385.83	1512.74
45	0	0	0	450	450	450	<1	<1	110.73	225.85
Average	0	0	0	280	280	280	0.1	0.11	1056.95	1139.24

**Table 3:** HS results for Tsiligirides 3.

*T*_*max*_	*r*	$\bar{e}$	$\bar{e_{l}}$	Best	*b*	*w*	$\bar{t}$	*σ*_*t*_	$\bar{it}$	*σ*_*it*_
15	0	0	0.00	170	170	170	<1	<1	4.93	10.30
20	0	0	0.00	200	200	200	<1	<1	113.77	248.15
25	0	0	0.00	260	260	260	<1	<1	2390.90	2081.08
30	0	0	0.00	320	320	320	<1	<1	110.43	328.51
35	0	0	0.00	390	390	390	<1	<1	638.33	840.32
40	0	0	0.00	430	430	430	<1	<1	674.33	971.62
45	0	0	0.00	470	470	470	<1	<1	139.10	213.47
50	0	0	0.00	520	520	520	<1	<1	390.87	402.38
55	0	0	0.00	550	550	550	<1	<1	284.17	456.47
60	0	0	0.00	580	580	580	<1	<1	201.20	301.25
65	0	0	0.00	610	610	610	<1	<1	108.87	222.20
70	0	0	0.00	640	640	640	<1	<1	1398.90	2047.87
75	0	0	0.00	670	670	670	1.11	1.52	4411.80	5884.41
80	0	0	**0.19**	710	710	710	6.58	7.93	24072.63	28986.59
85	0	0	0.00	740	740	740	1.23	<1	4318.27	3434.63
90	0	0	0.00	770	770	770	<1	<1	1391.70	1404.21
95	0	0	0.00	790	790	790	<1	<1	35.93	128.67
100	0	0	0.00	800	800	800	<1	<1	1.97	1.19
105	0	0	0.00	800	800	800	<1	<1	1.10	0.31
110	0	0	0.00	800	800	800	<1	<1	1.00	0.00
Average	0	0	**0.01**	561	561	561	0.52	0.62	2034.51	2398.18

**Table 4:** HS results for Tsiligirides 4.

*T*_*max*_	*r*	$\bar{e}$	$\bar{e_{l}}$	Best	*b*	*w*	$\bar{t}$	*σ*_*t*_	$\bar{it}$	*σ*_*it*_
5	0	0	0.00	10	10	10	<1	<1	1.70	3.83
10	0	0	0.00	15	15	15	<1	<1	1.33	1.83
15	0	0	0.00	45	45	45	<1	<1	100.97	185.99
20	0	0	0.00	65	65	65	<1	<1	68.33	178.01
25	0	0	0.00	90	90	90	<1	<1	1160.13	1604.71
30	0	0	0.00	110	110	110	<1	<1	56.10	140.26
35	0	0	0.00	135	135	135	<1	<1	429.77	631.25
40	0	0	0.00	155	155	155	<1	<1	4594.10	2864.82
46	0	0	0.00	175	175	175	<1	<1	317.70	515.95
50	0	0	0.00	190	190	190	<1	<1	242.07	360.82
55	0	0	0.00	205	205	205	6.33	6.26	30258.90	22.84
60	0	0	0.00	225	225	225	2.57	3.49	11625.23	15670.58
65	0	0	0.00	240	240	240	<1	<1	1405.70	1260.74
70	0	0	0.00	260	260	260	<1	<1	1325.50	2445.22
73	0	0	0.00	265	265	265	<1	<1	590.93	758.29
75	0	0	**1.03**	275	275	275	<1	<1	614.00	649.36
80	0	0	0.00	280	280	280	<1	<1	143.33	279.80
85	0	0	0.00	285	285	285	<1	<1	57.13	162.70
Average	0	0	**0.06**	168.06	168.06	168.06	0.61	0.67	2944.05	3207.61

**Table 5:** HS results for Chao 64.

*T*_*max*_	*r*	$\bar{e}$	$\bar{e_{l}}$	Best	*b*	*w*	$\bar{t}$	*σ*_*t*_	$\bar{it}$	*σ*_*it*_
15	0	0.00	0.00	96	96	96	<1	<11	18.27	92.12
20	0	0.00	0.00	294	294	294	<1	<1	1778.90	1227.85
25	0	0.00	0.00	390	390	390	<1	<1	526.10	680.96
30	0	0.00	0.00	474	474	474	5.64	7.77	14963.83	20719.07
35	0	0.00	0.00	576	576	576	3.42	2.27	8477.17	5648.80
40	0	0.00	0.00	714	714	714	1.55	1.77	3349.73	3977.60
45	0	0.00	0.00	816	816	816	16.29	17.79	26275.73	29187.47
50	0	0.00	**0.07**	900	900	900	24.75	25.63	35735.53	36973.19
55	0	**0.06**	**0.20**	984	984	**978**	163.32	203.36	213074.53	266271.87
60	0	0.00	**0.64**	1062	1062	1062	35.87	31.98	43432.80	38620.41
65	0	0.00	**0.59**	1116	1116	1116	19.5	19.42	21884.93	21896.12
70	0	0.00	**0.45**	1188	1188	1188	72.11	59.38	75625.67	62616.53
75	0	0.00	**0.42**	1236	1236	1236	58.61	68.84	59586.43	69379.58
80	0	0.00	**1.20**	1284	1284	1284	228.29	211.46	234803.23	220124.02
Average	0	0.00	**0.26**	795	795	794.57	44.99	46.44	52823.78	55529.69

**Table 6:** HS results for Chao 66.

*T*_*max*_	*r*	$\bar{e}$	$\bar{e_{l}}$	Best	*b*	*w*	$\bar{t}$	*σ*_*t*_	$\bar{it}$	*σ*_*it*_
5	0	0.00	0.00	10	10	10	<1	<1	1.00	0.00
10	0	0.00	0.00	40	40	40	<1	<1	9.43	43.93
15	0	0.00	0.00	120	120	120	<1	<1	1793.10	2197.55
20	0	0.00	0.00	205	205	205	<1	<1	6819.67	6866.79
25	0	0.00	0.00	290	290	290	1.14	1.24	5866.23	6437.46
30	0	0.00	0.00	400	400	400	<1	<1	2367.67	3140.48
35	0	0.00	0.00	465	465	465	<1	<1	1859.03	1924.74
40	0	0.00	0.00	575	575	575	1.77	2.05	5453.23	6163.61
45	0	0.00	0.00	650	650	650	7.11	11.90	14030.70	23312.01
50	0	0.00	0.00	730	730	730	2.50	2.30	4633.03	4314.21
55	0	0.00	0.00	825	825	825	3.55	3.60	5502.37	5551.85
60	0	0.00	0.00	915	915	915	3.03	2.07	4332.67	2981.41
65	0	0.00	0.00	980	980	980	4.23	2.57	5621.77	3462.94
70	0	0.00	0.00	1070	1070	1070	3.79	2.79	4661.27	3385.67
75	0	0.00	0.00	1140	1140	1140	4.41	3.86	5524.30	5019.51
80	0	0.00	**0.05**	1215	1215	1215	9.27	7.93	11556.03	9877.88
85	0	0.00	0.00	1270	1270	1270	10.39	8.25	11922.57	8926.66
90	0	0.00	**0.15**	1340	1340	1340	39.84	43.16	44948.23	49178.92
95	0	0.00	**0.07**	1395	1395	1395	43.87	38.37	47706.33	41499.41
100	0	**0.07**	**0.50**	1465	1465	**1455**	265.04	205.90	284238.33	218134.42
105	0	0.00	**0.70**	1520	1520	1520	201.36	188.02	202225.53	188550.24
110	0	**0.34**	**0.45**	1560	1560	**1550**	256.83	307.81	250348.10	300976.30
115	0	0.00	**0.44**	1595	1595	1595	63.11	47.37	59688.17	45000.14
120	0	**0.43**	**0.84**	1635	1635	**1625**	127.50	215.19	117531.83	198769.66
125	0	0.00	**0.97**	1670	1670	1670	50.89	42.45	45363.47	37967.62
130	0	0.00	**0.18**	1680	1680	1680	3.66	3.38	3145.37	2932.05
Average	0	0.03	**0.17**	952.31	952.31	951.15	42.51	43.94	44121.13	45254.44

**Table 7:** HS results for tasks from OPLib.

Task	*r*	$\bar{e}$	Best	*b*	*w*	$\bar{t}$	*σ*_*t*_	$\bar{it}$	*σ*_*it*_
att48	0.00	0.00	31	31	31	<1	0.04	91.00	218.92
gr48	0.00	0.00	31	31	31	<1	0.28	2250.87	1616.03
hk48	0.00	0.00	30	30	30	2.19	5.36	12863.37	30882.03
eil51	0.00	0.00	29	29	29	1.75	2.18	9192.90	11467.30
berlin52	0.00	0.00	37	37	37	33.50	50.78	135978.07	205707.10
brazil58	0.00	0.00	46	46	46	31.66	30.76	124574.13	118489.57
st70	0.00	0.00	43	43	43	7.87	13.79	20264.10	32810.85
eil76	0.00	1.06	47	47	**46**	109.90	128.44	207680.43	239188.59
pr76	0.00	0.41	49	49	**48**	86.90	120.93	149024.80	209310.03
gr96	0.00	1.04	64	64	**63**	204.50	296.82	194629.93	283407.94
rat99	0.00	1.03	52	52	**51**	105.75	138.02	136608.30	177893.11
kroA100	0.00	0.00	56	56	56	10.94	12.89	12984.10	14729.73
kroB100	0.00	0.06	58	58	**57**	261.06	310.50	243193.73	282625.61
kroC100	0.00	0.24	56	56	**54**	246.26	178.48	238541.10	171485.74
kroD100	0.00	1.47	59	59	**58**	79.15	145.94	77170.10	142296.40
kroE100	0.00	0.00	57	57	57	32.28	32.16	34303.50	34756.01
rd100	0.00	0.00	61	61	61	145.85	141.03	126609.20	122864.28
eil101	0.00	1.30	64	64	**63**	188.70	231.69	194471.17	238636.49
lin105	0.00	0.05	66	66	**65**	318.87	317.39	293713.37	284915.56
pr107	0.00	0.00	54	54	54	<1	0.01	1.40	0.81
gr120	1.33	2.53	75	74	**72**	565.89	459.57	365902.67	300722.09
pr124	0.00	0.00	75	75	75	61.72	70.18	47272.80	53896.16
bier127	0.00	0.71	103	103	**102**	812.69	922.11	220236.37	250993.28
pr136	1.41	2.58	71	**70**	**69**	457.25	471.20	211519.47	218866.95
gr137	0.00	0.86	81	81	**80**	555.63	576.56	253464.90	258844.67
pr144	0.00	0.00	77	77	77	90.94	75.34	54995.30	45769.43
kroA150	1.16	3.02	86	**85**	**82**	1261.70	956.83	432047.13	333513.37
kroB150	2.30	3.72	87	**85**	**82**	1356.18	710.60	474526.83	247806.30
pr152	1.30	2.08	77	**76**	**75**	586.10	512.26	322449.67	275730.26
u159	1.08	2.22	93	**92**	**90**	800.78	570.39	276149.17	193685.60
rat195	2.94	3.50	102	**99**	**97**	1472.35	1489.23	342144.87	342504.60
d198	1.63	2.38	123	**121**	**119**	1153.68	982.01	309872.67	269850.75
kroA200	3.42	4.76	117	**113**	**109**	2374.26	1440.44	409421.43	243507.06
kroB200	5.88	7.09	119	**112**	**109**	1791.61	1600.05	319671.63	279828.96
gr202	2.76	4.07	145	**141**	**138**	2970.64	2650.00	383818.93	341522.94
ts225	0.00	0.62	124	124	**121**	2934.53	1915.45	460920.50	300935.92
tsp225	4.65	6.05	129	**123**	**120**	2578.96	1248.38	431223.40	214970.62
pr226	3.17	3.99	126	**122**	**120**	1332.10	1049.04	321407.83	250433.02
gr229	3.41	5.00	176	**170**	**166**	5411.87	3837.91	446437.63	317779.69
gil262	6.96	7.93	158	**147**	**144**	3665.30	2193.72	472798.07	281641.66
pr264	0.00	0.00	132	132	132	6.92	7.38	1087.90	1200.15
a280	2.72	4.49	147	**143**	**139**	2893.90	1889.59	377433.23	250265.10
pr299	3.7	5.76	162	**156**	**151**	5897.06	3855.39	443727.50	296947.89
lin318	6.34	9.45	205	**192**	**183**	7850.21	5059.49	469143.27	295894.35
rd400	7.95	9.62	239	**220**	**214**	15925.15	9521.89	467329.10	282359.77
Average	1.42	2.20	89.31	87.18	85.47	1587.49	1027.17	233981.06	194506.06

For tasks from the Tsiligirides sets, the HS achieved the best solutions in all cases (both *r* and *e* amount to 0%), with the time to achieve convergence characterized by small values (for the vast majority of analysed values *T*_*max*_, $\bar{t}$ amounted to less than one second), indicating the problem-free possibility of applying the method to solve practical problems with characteristics that align with the instances analyzed. Meanwhile, analyzing $\bar{it}$ and *σ*_*it*_ confirm the possibility of limiting the value of *IT* while maintaining the quality of the solutions constructed.

For bigger instances of the OP problem, from the sets of Chao 64 and 66, the HS usually obtained the best solutions. However, for some values *T*_*max*_ (55 for Chao 64 and 100, 110 and 120 for Chao 66), suboptimal route plans were also constructed, characterized by slightly worse objective function value (acceptable for many practical applications). The time required to achieve convergence $\bar{t}$ increased to more than 40 seconds on average. Additionally, the number of iterations required to achieve convergence by the technique increased. These observations may indicate the need to improve the method, through a better choice of value of the parameters or replacement of the local search technique (2-opt) with the algorithm characterized by better efficiency.

**Fig 7:**
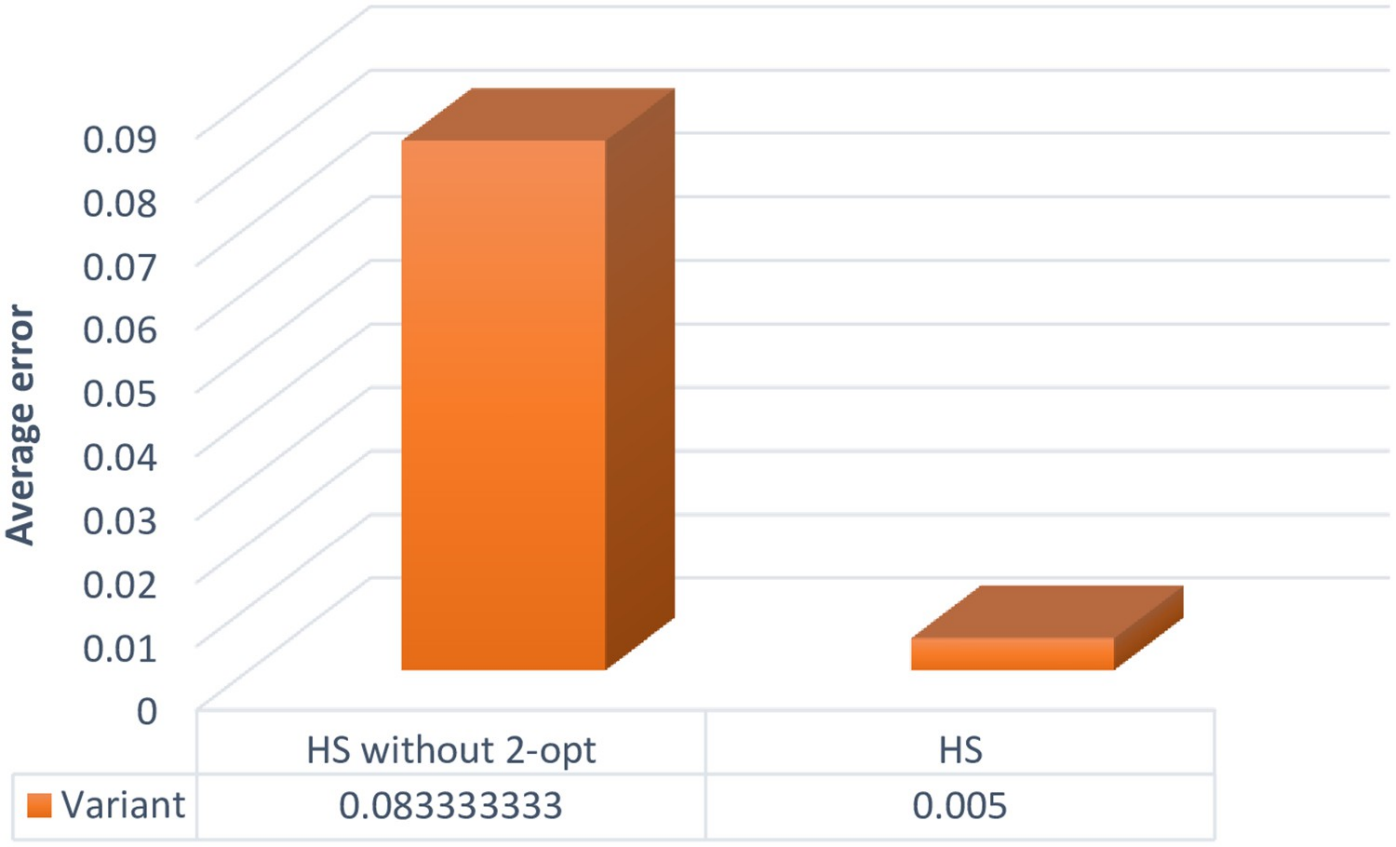
Comparison of the average error for the tasks from the Tsiligirides and Chao sets achieved by the HS and HS without 2-opt.

Based on the comparison of the average error for the tasks from the Tsiligirides and Chao sets achieved by the HS and HS without 2-opt (presented in Fig 7) and Wilcoxon test results (shown in the Table 8), significant effectiveness of the hybrid approach was found and its use is recommended.

**Table 8:** Results of Wilcoxon Signed-Rank Test for the conducted research.

*V*2	HS	HS without 2-opt
*V*1		
HS	N/A	**9.46475E-35**
HS without 2-opt	1	N/A

Tasks derived from OPLib are characterized by different sizes, enabling the determination of the behavior of the proposed algorithm for different OP instances. For tasks consisting of fewer nodes than 150, very high HS efficiency can be observed (only for two tasks out of 26 no optimal solution was designated). However, as the number of nodes increased, the algorithm began to obtain more and more near-optimal results, which indicates the need to adjust the HS parameter values for larger instances. Both the average error obtained and the time to achieve convergence testify to the possibility of using HS to solve many practical problems.

Table 9 compares the HS results with the results presented in the literature on the subject, revealing that the proposed algorithm is much more efficient and can compete with the best state-of-the-art methods. The HS achieved the most advantageous average value *w* for Chao 64, with less beneficial results only observed for Chao 66 (the average value *w* was worse than the average values achieved by both for the ACO algorithm and the GRASP with PR approach).

**Table 9:** Comparison of HS results with results presented in the subject literature.

Method	Tsiligirides 1		Tsiligirides 2		Tsiligirides 3		Tsiligirides 4		Chao 64		Chao 66	
	Average *b*	Average *w*	Average *b*	Average *w*	Average *b*	Average *w*	Average *b*	Average *w*	Average *b*	Average *w*	Average *b*	Average *w*
HS	**167.78**	**167.78**	**280.00**	**280.00**	**561.00**	**561.00**	**168.06**	**168.06**	**795.00**	794.57	**952.31**	951.15
D algorithm	-	-	179.09	179.09	390.50	390.50	-	-	-	-	-	-
S algorithm	166.11	166.11	272.27	272.27	487.00	487.00	-	-	-	-	-	-
center-of-gravity	160.28	160.28	272.27	272.27	552.50	552.50	-	-	-	-	-	-
CGW	**167.78**	**167.78**	**280.00**	**280.00**	**561.00**	**561.00**	167.78	167.78	790.71	790.71	948.65	948.65
GA	**167.78**	**167.78**	**280.00**	**280.00**	**561.00**	**561.00**	**168.06**	**168.06**	-	-	-	-
GLS	163.89	163.89	275.91	275.91	558.50	558.50	-	-	786.00	786.00	942.88	942.88
ACO	**167.78**	**167.78**	**280.00**	**280.00**	**561.00**	**561.00**	-	-	**795.00**	792.00	**952.31**	**952.31**
VNS	**167.78**	**167.78**	**280.00**	**280.00**	**561.00**	**561.00**	-	-	**795.00**	790.71	**952.31**	947.50
IS-PSO	-	-	**280.00**	270.91	**561.00**	549.50	-	-	-	-	-	-
GRASP	-	-	-	-	-	-	-	-	794.57	791.14	**952.31**	948.27
GRASP with PR	-	-	-	-	-	-	-	-	**795.00**	792.43	**952.31**	**952.31**
GSOA	-	-	-	-	-	-	-	-	775.72	760.14	927.88	906.46

Comparison of results obtained by HS and state-of-the-art algorithms for tasks from OPLib is presented in Table 10 (optimal results are in bold). On their basis, we found significant efficiency of our approach, which gained an advantage over 2-Parameter IA and EA4OP for tasks, characterized by the occurrence of up to 144 nodes. For larger tasks, the method obtains slightly worse results than competing approaches, but this difference could be eliminated by further improving the technique (in this paper we focused on choosing parameter values so that the algorithm copes well with tasks of moderate size). It is worth noting that the average objective function value for HS was higher than for the 2-Parameter IA.

**Table 10:** Comparison of HS results with results from subject literature for tasks from OPLib.

Task	HS	2-Parameter IA	GRASP with PR	EA4OP
att48	**31**	**31**	**31**	**31**
gr48	**31**	**31**	**31**	**31**
hk48	**30**	**30**	**30**	**30**
eil51	**29**	**29**	**29**	**29**
berlin52	**37**	**37**	**37**	**37**
brazil58	**46**	**46**	**46**	**46**
st70	**43**	**43**	**43**	**43**
eil76	**47**	46	**47**	46
pr76	**49**	**49**	**49**	**49**
gr96	**64**	**64**	**64**	**64**
rat99	**52**	51	**52**	**52**
kroA100	**56**	**56**	**56**	55
kroB100	**58**	**58**	**58**	57
kroC100	**56**	**56**	**56**	**56**
kroD100	**59**	**59**	**59**	58
kroE100	**57**	55	**57**	**57**
rd100	**61**	**61**	**61**	**61**
eil101	**64**	63	**64**	**64**
lin105	**66**	**66**	**66**	**66**
pr107	**54**	**54**	**54**	**54**
gr120	74	74	**75**	74
pr124	**75**	**75**	**75**	**75**
bier127	**103**	**103**	**103**	**103**
pr136	70	69	70	**71**
gr137	**81**	**81**	**81**	78
pr144	**77**	73	**77**	**77**
kroA150	85	85	**86**	**86**
kroB150	85	86	86	86
pr152	76	76	**77**	**77**
u159	92	82	92	92
rat195	99	99	99	99
d198	121	120	122	**123**
kroA200	113	112	**117**	**117**
kroB200	112	117	118	**119**
gr202	141	140	**145**	**145**
ts225	**124**	**124**	**124**	**124**
tsp225	123	117	126	127
pr226	122	121	**126**	**126**
gr229	170	174	174	**176**
gil262	147	150	151	156
pr264	**132**	**132**	**132**	**132**
a280	143	133	143	143
pr299	156	154	158	160
lin318	192	194	200	202
rd400	220	218	225	234
Average	87.18	86.53	88.27	88.62

## 7 Conclusions and further research plans

The proposed modifications enabled the design of an efficient HS variant that achieved solutions characterized by an average error below the 0.01% level for the classic benchmark. For all 107 OP instances—derived from the six most-popular sets of tasks—the proposed approach was able to construct optimum route plans that, along with the short time of reaching convergence and favorable results when compared with the state-of-the-art methods, confirm the possibility of using the prepared algorithm in economic practice.

For the tasks from OPLib, our algorithm has proved to be highly effective, in particular for tasks with fewer nodes than 150 (it obtained better results than 2-Parameter IA and EA4OP). For larger tasks, the method obtains slightly worse results than competing approaches, but this difference could be eliminated by further improving the technique.

Future work on this subject should check the efficiency of the HS in the context of using other local search techniques (other than 2-opt; e.g. 3-opt, Lin–Kernighan heuristic [46] or Lin–Kernighan–Helsgaun algorithm [47]) and places and criteria (e.g., allowing improving of slightly worse solutions from the worst *HM* harmony) of its occurrence is recommended (studies on the influence of the localization of the local search algorithm in HS are presented in the paper [41]). Furthemore, additional tests should be performed, including the assignment of optimal values of algorithm parameters and checking the effectiveness of the method on other tasks from OPLib. Finally, it would be worthwile adjusting the method to address extended OP variants (e.g., OP with time windows [48] and TOP [49]).
